# Sarcoidosis and Work Participation: The Need to Develop a Disease-Specific Core Set for Assessment of Work Ability

**DOI:** 10.1007/s00408-019-00234-3

**Published:** 2019-05-17

**Authors:** C. M. R. Hendriks, L. A. Saketkoo, M. D. P. Elfferich, J. De Vries, P. A. H. M. Wijnen, M. Drent

**Affiliations:** 1 ILD Care Foundation Research Team, Ede, The Netherlands; 2 Faculty of Medicine, Utrecht University, Utrecht, The Netherlands; 3 ILD Center of Excellence, Department of Pulmonology, St. Antonius Hospital, Koekoekslaan 1, 3435 CM Nieuwegein, The Netherlands; 4 Divisions of Pulmonary Medicine and Rheumatology, Tulane University School of Medicine, New Orleans, LA USA; 5 New Orleans Scleroderma & Sarcoidosis Patient Care & Research Center, New Orleans, LA USA; 6 UMC Comprehensive Pulmonary Hypertension Center, New Orleans, LA USA; 7 Department of Medical Psychology, ETZ (Elisabeth-TweeSteden Hospital) Tilburg, Tilburg, The Netherlands; 8 Department of Medical and Clinical Psychology, Tilburg University, Tilburg, The Netherlands; 9 Department of Clinical Chemistry, Central Diagnostic Laboratory, Maastricht University Medical Center, Maastricht, The Netherlands; 10 Department of Pharmacology and Toxicology, Faculty of Health, Medicine and Life Science, Maastricht University, Maastricht, The Netherlands

**Keywords:** Work ability, Absenteeism, Disability, Sarcoidosis, Fatigue, Small fiber neuropathy-associated symptoms

## Abstract

**Objective:**

Sarcoidosis, an inflammatory multi-organ disease with a wide variety of clinical manifestations, affecting people of working age. Patients suffer from a broad spectrum of physical symptoms of varying severity that impact function including cognitive impairment and disabling fatigue. The Dutch Sarcoidosis Society identified a knowledge gap in various facets related to work ability. The aim of this study was to assess sarcoidosis patients’ perceived problems related to work performance, employer, and disability evaluations.

**Methods:**

A cross-sectional web-based anonymous survey was conducted among Dutch sarcoidosis patients recruited through sarcoidosis patient societies and outpatient sarcoidosis clinics. This investigation queried work performance, employer support, and disability evaluations.

**Results:**

The study sample included 755 patients of whom 43% (*n* = 328) had undergone disability evaluation and were significantly more likely to experience extrapulmonary symptoms, severe fatigue, reduced exercise capacity along with memory problems and concentration problems with higher mean FAS and SFNSL-scores. Of these 328, 37% (*n* = 121) perceived they had not been listened to or taken seriously at assessments, and 38% (*n* = 124) disagreed with the outcome of disability assessments by benefits authorities; 75% (*n* = 93) appealed or requested re-assessment.

**Discussion:**

A better understanding of sarcoidosis-related impact on work ability and quantification of disease burden is needed. Education for medical examiners and employers on sarcoidosis may improve quality of assessments and work accommodations. Development of guidelines for benefit authorities, which consider the broad impact of sarcoidosis beyond that of reduced pulmonary function, including extra-pulmonary assessment like fatigue, cognitive difficulties, as well as other organ involvement are needed.

## Background

Sarcoidosis generally occurs among the relatively young, working population. Sarcoidosis is a heterogeneous multi-organ system disease whereby the immune system launches a response to an unknown antigen resulting in granulomatous lesions occurring most anywhere in the body interfering with resident organ function. The lungs, eyes, and skin are the most commonly recognised; but cardiac, brain, spinal cord, and hepatic involvement are not uncommon [1].

Depending on granuloma burden, location, and sensitivity of the organ involved (e.g. eye, heart, brain and spinal cord require little granuloma volume to have devastating clinical effects); symptoms related to sarcoidosis vary in type, severity, and extent of disability [2]. Apart from major organ involvement, sarcoidosis can involve reduced muscle strength, loss of physical condition, pain, extreme fatigue, and memory- and concentration problems. Disease chronicity correlates to higher number and degree of reported impairment [3, 4].

These impairments can be organ-related, such as dyspnoea or exercise intolerance related to pulmonary or cardiac involvement; but might also be non-organ related, non-specific. Apart from major organ involvement, sarcoidosis can involve reduced muscle strength, loss of physical condition, pain, extreme fatigue, and small fiber neuropathy (SFN)-associated symptoms. Psychological factors are pervasive including anxiety, concentration and memory difficulties, and depressive symptoms; and may not correspond with inflammatory disease activity nor respond to sarcoidosis treatment [5, 6]. These symptoms are disabling, persisting after other signs of sarcoidosis activity resolve, and adversely impact major life areas, including quality of life (QOL) and work ability [7, 8].

Sarcoidosis is associated with a high number of illness and health visit related sick-days and associated with large yearly income loss [9–11] that persists beyond 5 years from diagnosis [9]. Patients undergo mental and physical employment assessments that depend upon lung function as the main indicator for work capacity, despite sarcoidosis being a systemic multi-organ disease with many patients having severe disease without significant lung impairment [6, 12–20]. The Dutch Sarcoidosis society (www.sarcoidose.nl) [21] reported a need for educational enhancement of sarcoidosis among decision-making authorities and medical examiners performing work capacity assessments in sarcoidosis patients, particularly in regard to extent and severity of extra-pulmonary symptoms.

The aim of this study was to assess difficulties sarcoidosis patients may have experienced regarding their work including performance, absenteeism, environment, employer policies, and disability evaluations. These experiences were examined in relation to disease burden.

## Methods

### Study Design

In cooperation with the Dutch Sarcoidosis Society, Sarcoidose.nl [21] and the ILD care foundation, the authors designed a cross-sectional web-based anonymous questionnaire that broadly investigated potential work-related issues experienced by patients with sarcoidosis in correlation to symptom and disease burden. Recruitment occurred from October 2017 to April 2018 and was designed to engage large representative samples of sarcoidosis patients.

### Study Subjects and Procedure

Patients were recruited through membership of the Dutch Sarcoidosis Society, Sarcoidose.nl [21] via the society’s newsletter and advertisement through ILD Center of Excellence at Nieuwegein. No incentives were offered. Patients participating were proficient in Dutch and had internet access.

Patients were provided the specific link to the survey through the online questionnaire tool *Surveymonkey* (www.surveymonkey.com) [22] which queried disease and symptom burden, experiences regarding employers and disability evaluations, as well as demographics (gender, age, disease duration), medication use, and two sarcoidosis-validated questionnaires; the Fatigue Assessment Scale (FAS) [23] and the Small Fiber Neuropathy Screening List (SFNSL) [24].

### Questionnaires

The FAS is a ten-item self-report fatigue questionnaire rated on a five-point scale (1 never to 5 always) providing a total score ranging between 10 and 50, and mental and physical sub-scores. A score > 21 indicates fatigue with > 34 indicating extreme fatigue. The FAS demonstrated good reliability and validity in sarcoidosis [23].

The SFNSL is a 21-item self-administered screening questionnaire for SFN-related symptoms on a five-point scale (0 never to 4 always). Scores range from 0 to 84, with scores between 11 and 48 indicating probable to highly probable SFN and > 48 indicating SFN [24].

### Statistical Analysis

All statistical analyses were performed using SPSS version 20 for Windows. Standard descriptive statistics were computed. After subdivision into assessed- and non-assessed for disability benefits, these two sarcoidosis patient samples were compared using Chi-square test and independent *t *tests, depending on type of variables (continuous or dichotomous). A probability (*p*) value of < 0.05 was considered to be statistically significant.

## Results

**Fig. 1:**
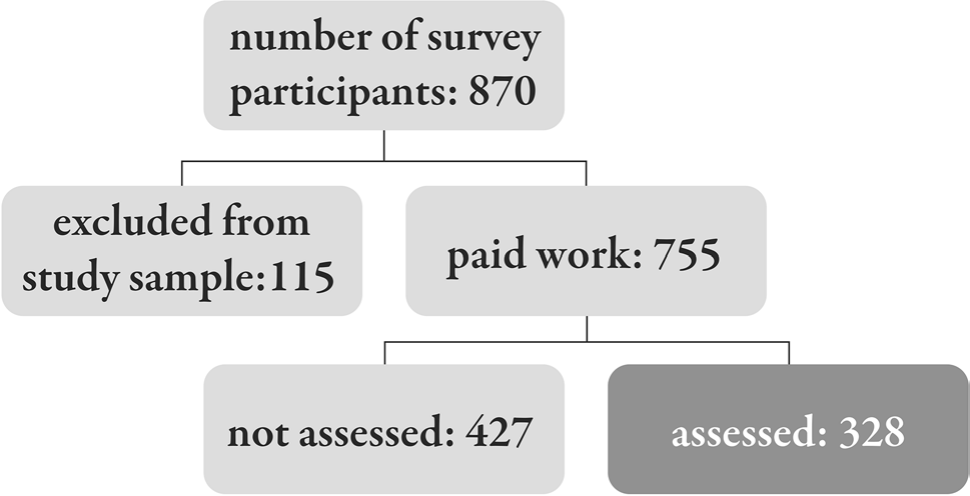
Flowchart of survey participants

Of the 870 patients with sarcoidosis who participated in the survey, 755 had paid work. Of these 755, 43% (*n* = 328) reported having undergone disability assessments by the benefits authorities; 427 patients reported no assessment (Fig. 1; Table 1). The remaining 115 patients were not included due to being homemakers, informal caregivers, self-employed, or previously declared unfit for work due to illnesses other than sarcoidosis.

**Table 1:** Characteristics of patients with sarcoidosis, who were assessed or not assessed by the benefits authority

	Assessed	Not assessed	*p*-Value
Number	328	427	
Gender (male, %)	49	50	0.750
Age (years, mean ± SD)	51.8 ± 8.9	50.4 ± 10.2	0.046
Symptoms^a^			
None (%)	1	2	0.132
Organ-related (%)	97	94	0.041
Pulmonary (%)	72	67	0.162
Extrapulmonary (%)	95	85	< 0.001
Not organ-related (%)	99	94	0.001
Fatigue (%)	97	88	< 0.001
Reduced exercise capacity (%)	92	78	< 0.001
Concentration problems (%)	69	47	< 0.001
Memory problems (%)	30	21	0.009
Medication			
None (%)	24	36	0.001
Questionnaires			
Mean FAS-score ± SD	36 ± 7.3	31 ± 8.4	< 0.001
FAS-score classification			
Non-fatigue (based on FAS-score) (%)	5	15	< 0.001
Fatigue (based on FAS-score) (%)	35	49	< 0.001
Extreme fatigue (based on FAS-score) (%)	61	36	< 0.001
Mean SFNSL-score ± SD	34 ± 21.1	23 ± 15.2	< 0.001
SFNSL-score classification			
No SFN (%)	11	24	< 0.001
(Highly) probable SFN (%)	62	69	< 0.001
SFN (%)	27	6	< 0.001

^a^For further explanation of the variables see “Appendix” section

Of those assessed, 75% were on treatment compared to almost two-thirds of the non-assessed sample (*p* < 0.01), and also had significantly more extra-pulmonary symptoms, extreme fatigue, reduced exercise capacity, and memory and concentration problems. Moreover, mean FAS and SFNSL-scores were significantly higher in the assessed group.

At the time of the survey 17% of the total study sample were on sick leave, 28% were declared fully unfit for work, and 13% declared partially unfit for work. Among those employed (*n* = 267) at the time of the survey, 69% reported frequently finding work too strenuous, and reported sarcoidosis-related absence for shorter or longer periods. Among those assessed (*n* = 328), 37% perceived not having been listened to or taken seriously during assessment, while 10% had mixed experiences. Thirty-eight percent (*n* = 124) of those assessed disagreed with the assessment outcome; of which 75% (*n* = 93) appealed or requested re-assessment. Table 2 summarises the respondents’ perceptions of the assessment encounter [25].

**Table 2:** Ten statements by patients with sarcoidosis, who had been assessed by the Dutch benefits authority UWV

“I was constantly being treated as if I didn’t WANT to work, while in my case (and no doubt for most people who are ill) the problem is actually that I want to work so much that I tend to underestimate my illness.”
“What I failed to find at UWV was a humane attitude. I felt I was being dismissed as the umpteenth person who came to claim benefit.”
“The medical examiner ought to pay more attention to a patient’s personal situation; for instance, I was told I could become a typist, even though I have problems with my hands due to small fiber neuropathy and I’m also dyslexic. It totally beats me how they could arrive at such a conclusion, but the UWV regards it as completely rational.”
“According to the UWV doctor, extreme fatigue due to sarcoidosis is not a sufficient argument for declaring me largely unfit for work. You’ re supposed to spend whatever energy you have on work. How you cope at home is not their problem.”
“The UWV doctor made light of my symptoms, as my lung function is still good.”
“The UVW doctor said to me in a sarcastic tone that our king Willem-Alexander had also had it, and he had fully recovered.”
“The entire process took many years; years of great tension and not being taken seriously. ‘Fortunately’, I got, during those years, more and more physical complaints, she spoke cynically…”
“Company doctors and medical examiners should know more about sarcoidosis, so they understand us better. The problem with sarcoidosis is that you never know when it’s going to strike: the fatigue and dyspnoea are always just round the corner and that’s what makes the future so insecure. Sarcoidosis is unpredictable and is characterized by good and very bad days.”
“The contacts between the insurance medical examiners and your specialist should be improved and take less time. The lines should be shorter!”
“There’s a need for better explanation of the pathway that will be followed, how the assessments work.”

UWV: Dutch Employee Insurance Agency, which is an autonomous administrative authority and is commissioned by the Ministry of Social Affairs and Employment (SZW) to implement employee insurances, such as the WIA (Work and Income according to Labour Capacity Act), which contains the IVA (Full Invalidity Benefit Regulations) and the WGA (Return To Work (Partially Disabled) Regulations [25]

## Discussion

This is the first study assessing difficulties sarcoidosis patients may have experienced regarding work capacity in relation to disability claims. Sarcoidosis patients who had undergone evaluation for disability were significantly more likely to experience extra-pulmonary symptoms, extreme fatigue, reduced exercise capacity, and cognitive difficulties.

At the time of the survey, a high rate of participants were on sick leave, or declared fully or partially unfit for work demonstrating the great impact of sarcoidosis on work ability. Moreover, a remarkably high proportion of assessed patients perceived they were not taken seriously or listened to during assessments resulting in a high level of outcome disagreement, suggesting the need to improve quality of assessments better informed by the scope of sarcoidosis.

### Absenteeism Related to Sarcoidosis

Sarcoidosis-related absenteeism has previously been demonstrated to impair work attendance and livelihood [9–11], imposing significant economic burden on both patients and employers. Polish social insurance data reported sarcoidosis-related absence or disability averaged 30 sick-days per person annually [11]. In the U.S. Sarcoidosis patients had significantly more sick-days (15.9 vs. 11.3) and income loss than non-sarcoidosis controls [10]. In Sweden, impaired sarcoidosis-related work ability persisted beyond 5 years after diagnosis, averaging 45 sick-days annually compared with 34 days in non-sarcoidosis disease comparators. Older patients and patients receiving treatment upon diagnosis registered the highest number of sick-days and largest income loss during the study period [9].

Combining results from our and these few other studies, as most patients want to continue working, attention on the causes of absenteeism and strategies to improve work accommodations is needed. Also, health systems approaches that consolidate travel to clinical appointments and use home-based therapies as well as innovative applications such as sick-day donation and work from home infrastructures are worth taking a closer look at [26–30].

Beyond optimisation of work maintenance, disability assessments for sarcoidosis should be conducted by examiners well-educated in the scope of the disease.

### Assessing Claims and Collecting Information

The first step in evaluating claims for work capacity is data collection of diagnoses, physical examinations, laboratory findings, workload, and self-evaluation using questionnaires, performance tests, and interview procedures. Evaluation of disability is a complex process that is affected by the skills set, attitudes and beliefs of the examiner; few countries enforce standards of practice [31, 32], which presents considerable challenges to reliability of assessments [33, 34].

A recent systematic review by Barth et al. [35] supported this hypothesis demonstrating a high variation in judgements on work disability among medical examiners; using standardised assessment methods, like rating instruments for functional limitations or structured interview methods (such as the disability assessment structured interview (DASI) [36]), demonstrated a reduction in “inter-rater variability” and improved the reproducibility among examiners.

However, only few of these standardised methods are available and being used to measure impairments in claim assessments. The aforementioned systemic review demonstrated that only six of thirteen insurance-based studies administered one or more specific work-ability instruments; with no instrument reported as validated. Most studies (*n* = 10) used only medical examiner perceptions to generate a global rating of workability [35].

In our study, a large percentage felt their complaints were not taken seriously. One could speculate that medical examiners erroneously regard sarcoidosis as a pulmonary disease, basing functional capacity mainly on the presence of pulmonary symptoms and lung function tests results. Recent research by Marcellis et al. [16] and Strookappe et al. [17] showed that generally used medical assessments, like lung function tests results and chest radiographs, only poorly correlate with commonly reported physical impairments such as muscle weakness, exercise intolerance, and fatigue.

This suggests that the inclusion of metrics such as 6-minute walk distance (6MWD), or other muscle and exercise testing in assessment guidelines may increase the accuracy of these assessment outcomes. A recent patient survey reinforced the need for clinical indicators to be used in tandem with patient-centred healthcare, including attention to work ability and other supportive measures [7].

### Knowledge About Symptom Burden in Sarcoidosis

Furthermore, this discordance underscores the limited knowledge that clinicians generally possess regarding sarcoidosis as a disease of extra-pulmonary involvement. Fatigue is a central concern in sarcoidosis [6, 15], our data demonstrated 35% and 61% with fatigue and extreme fatigue respectively in the assessed group; mirroring that found in many other chronic disorders [37–41]. Furthermore, sarcoidosis patients often struggle with memory and concentration problems [6, 20] (30% and 69% respectively in the assessed group), although these factors pose challenges in objective assessment, they are recognised illness-related symptoms meriting formal consideration with validated instruments like the FAS [23], the SFNSL [24] and the subjective Cognitive Failure Questionnaire (CFQ) [42].

Sarcoidosis patient organisations are an immense and rich resource to provide integral guidance on and development of effective methods of measuring and incorporating QoL and functionality outcomes with a view to improve sarcoidosis outcomes and management strategies [7]. In line with recent studies [7, 43], the results of this study suggest an urgent need for education among medical examiners supported by formal guidelines that consider all aspects of sarcoidosis.

The development of a disease specific core set for sarcoidosis under the framework of “the International Classification of Functioning, Disability and Health (ICF)” [44] with input from patients as well as physicians, rehabilitation specialists, specialist nurses, and so on, could hereby be a first step in examining and structuring the multi-faced functional impact of sarcoidosis on employment. As a preliminary step, this study investigated the merit of pre-study concerns expressed by the sarcoidosis patient community regarding experiences and perceptions of disability claims. This paper establishes that merit. Evaluation of disability is a complex process that is affected by the skills set, attitudes and beliefs of the examiner, which presents considerable challenges to the reliability of disability claims assessments. Future goals in addition, to correlation of disability claims with objective disease severity assessments, would investigate the spectrum of reliable and feasible evaluation measures to accurately quantify extent of sarcoid-related disability and investigate perspectives of disability officers in relation to sarcoidosis. The synthesis of these investigative efforts will ultimately help to inform the revision of national disability assessment protocols. The results of this paper once again highlight that non-pulmonary symptoms and measures should be acknowledged in the management of sarcoidosis in general, and more specifically by those who are responsible for rating workability.

## Limitations

A major limitation of the study might be selection bias with symptomatic patients being more likely to be members of a patients’ society or referred to a sarcoidosis clinic, and thus possibly influencing the rate of symptomatic respondents. To date, we recently published a study evaluating self-reported symptoms in three European Countries: Denmark, Germany, and the Netherlands whereby the German and Dutch cohorts were gathered through patient associations, the Danish cohort were patients from a sarcoidosis clinic. The German and Dutch cohorts had similar patient-reported severity as the Danish cohort, that had detailed objective severity data. This finding provides some reassurance regarding the Netherlands self-reported patient association data in this study [45].

Another limitation is that data is self-reported and without verification of diagnosis nor correlation to other objective functional measures. Having hospital established severity data would have enhanced the design—but this is a preliminary study that founds the need for more involved studies. However, symptom burden and perceptions of disability evaluations processes are both intrinsically patient experiences and never before quantified.

Although this study was based on self-reported symptoms and data on functional status was not collected, it is clear patients are plagued by multiple symptoms, most prominently fatigue. Earlier studies identified discordance between fatigue and lung function testing or 6MWD.

The present study only scratches the surface of many important aspects of work ability, setting the stage for future studies delving into work accommodations, clinical operations and disability assessments [46]. Future studies will correlate other functional testing to these patient-reported measures in relation to work capacity.

## Conclusion

Sarcoidosis patients may be more severely disabled than current disability claims assessment protocols for sarcoidosis are equipped to measure; a sufficient extent of objective parameters appear to be lacking. This study showed that a high proportion of the sarcoidosis patients who had undergone a disability evaluation felt their concerns dismissed with many disagreeing with the assessment outcome. Medical examiners’ lack of education regarding scope of sarcoidosis disease burden beyond being a pulmonary disease could explain these perceptions. There is an urgent need for sarcoidosis-related education enhancing work-related medical examinations supported by guidelines that account for extent of sarcoidosis impact on functionality, and therefore work capacity.

## Data Availability

The datasets used and/or analysed during the current study are available from the corresponding author on reasonable request.

## Appendix

Description variables Table 1.

**Table:**

Organ-related symptoms	Not organ-related symptoms
Pulmonary symptoms Cough Dyspnea	Fatigue Pain Reduced exercise capacity Concentration problems Memory problems Sleep problems
Extra-pulmonary symptoms Joint complaints Muscle complaints Skin abnormalities Cardiac arrhythmias Fainting Dry or running eyes Uveitis Macula edema Reduced vision Neurosarcoidosis-associated symptoms Liver function disorders Renal dysfunction Kidney stones Hypercalcemia Vitamin D deficiency Restless legs Dizziness	
